# Water Body Extraction Methods for SAR Images Fusing Sentinel-1 Dual-Polarized Water Index and Random Forest

**DOI:** 10.3390/s25154868

**Published:** 2025-08-07

**Authors:** Min Zhai, Huayu Shen, Qihang Cao, Xuanhao Ding, Mingzhen Xin

**Affiliations:** 1 College of Geodesy and Geomatics, Shandong University of Science and Technology, Qingdao 266590, China; zhaimin@sdust.edu.cn (M.Z.); shenhuayu@sdust.edu.cn (H.S.); 19811823821@163.com (Q.C.); 18263330609@163.com (X.D.); 2 Key Laboratory of Beidou Navigation and Intelligent Spatial Information Technology Application, Shandong University of Science and Technology, Qingdao 266590, China; 3 Key Laboratory of Ocean Geomatics, Ministry of Natural Resources of China, Qingdao 266590, China

**Keywords:** Sentinel-1, SDWI, RF, NDWI, water body extraction

## Abstract

Synthetic Aperture Radar (SAR) technology has the characteristics of all-day and all-weather functionality; accordingly, it is not affected by rainy weather, overcoming the limitations of optical remote sensing, and it provides irreplaceable technical support for efficient water body extraction. To address the issues of low accuracy and unstable results in water body extraction from Sentinel-1 SAR images using a single method, a water body extraction method fusing the Sentinel-1 dual-polarized water index and random forest is proposed. This novel method enhances water extraction accuracy by integrating the results of two different algorithms, reducing the biases associated with single-method water body extraction. Taking Dalu Lake, Yinfu Reservoir, and Huashan Reservoir as the study areas, water body information was extracted from SAR images using the dual-polarized water body index, the random forest method, and the fusion method. Taking the normalized difference water body index extraction results obtained via Sentinel-2 optical images as a reference, the accuracy of different water body extraction methods when used with SAR images was quantitatively evaluated. The experimental results show that, compared with the dual-polarized water body index and the random forest method, the fusion method, on average, increased overall water body extraction accuracy and Kappa coefficients by 3.9% and 8.2%, respectively, in the Dalu Lake experimental area; by 1.8% and 3.5%, respectively, in the Yinfu Reservoir experimental area; and by 4.1% and 8.1%, respectively, in the Huashan Reservoir experimental area. Therefore, the fusion method of the dual-polarized water index and random forest effectively improves the accuracy and reliability of water body extraction from SAR images.

## 1. Introduction

Surface water bodies are crucial components of the earth, playing a vital role in maintaining ecosystem balance and serving as an essential resource for human survival [1,2]. With the intensification of global climate change and the continuous enhancement of human activities, a series of negative impacts related to water resources have been triggered, such as water resource shortages, flood disasters, and pollution [3]. Therefore, strengthening real-time monitoring and early-warning systems for water resources and improving the accuracy and timeliness of water body information are of great significance for water resource management, ecological protection, and research on global water resource change [4,5].

With the application of high-resolution satellite imagery, water body extraction tends to be refined; using the comprehensive method of spectral, spatial structure, and texture feature analysis significantly improves extraction accuracy [6]. Optical remote sensing data, with their rich spectral information, short revisit cycles, and high spatial resolution [7], provide crucial support for research in land cover classification and water body extraction [8]. At present, water extraction methods based on optical imagery mainly include the threshold method [9,10] and the water index method [11]. Among them, the water index method can effectively use the spectral characteristics of water in a specific band, highlight information about the water, and reduce the influence of other ground objects, so that the water extraction precision is higher; the operation is relatively simple, and it has become a research hotspot. Inspired by the Normalized Difference Vegetation Index (NDVI), McFeeters [12] proposed the Normalized Difference Water Index (NDWI), which utilizes differences in spectral band reflectance to identify water bodies. Xu [13] improved upon the NDWI by replacing the near-infrared band with the shortwave infrared band from Thematic Mapper (TM) imagery, resulting in the Modified Normalized Difference Water Index (MNDWI). Buma et al. [14], by comparing several water body indices, found that MNDWI can effectively suppress vegetation and build noise through the short-wave infrared band, enhancing the spectral characteristics of water bodies. Feyisa et al. [15] proposed a new automated water body extraction index (the Automated Water Extraction Index, AWEI), which effectively improves the accuracy of water body mapping using optical images.

However, optical sensors are prone to interference under adverse conditions such as cloud cover and rainy weather, leading to observation interruptions or data loss, which limits their application in continuous monitoring and emergency response [16]. In contrast, microwave remote sensing, especially Synthetic Aperture Radar (SAR) technology, offers all-weather and all-day observation capabilities. It is not affected by weather or lighting conditions and can provide high-quality imagery under complex meteorological conditions [17]. A notable feature of SAR imagery is the limited number of bands, but the backscatter coefficient, texture features, and surface contours of objects play crucial roles in the extraction process [18]. The information in a radar image is obtained through the interaction with the surface signal, which reflects the backscattering amount of the ground target. This backscattering is closely related to surface roughness and dielectric properties. The surface of a water body is relatively smooth, usually manifested as a specular reflection, so it presents a low backscattering coefficient in a radar image, making the signal received by the sensor weak, and the image is smooth with dark characteristics overall [19]. However, in contrast, non-water body areas (such as land, vegetation, buildings, etc.) scatter radar waves in all directions due to high surface roughness and have a high backscattering coefficient, presenting a brightly colored area on a radar image, in sharp contrast to the dark area presented by water [20,21]. Therefore, for SAR images, especially the high-resolution data from Sentinel-1, this method is capable of effectively distinguishing between water and non-water areas, providing high-precision water extraction and monitoring results. This makes it of significant value in fields such as water resource management and environmental monitoring.

Water body extraction methods based on SAR images mainly include the random forest (RF) algorithm and water index methods. RF is a machine learning algorithm based on an ensemble of decision trees. It can perform automatic classification and extraction of water body areas by being trained on multiple features of SAR images, such as backscatter coefficients, texture features, and polarization information. Du et al. [22] employed two advanced ensemble learning classifiers, RF and Rotation Forest, to classify fully polarimetric SAR images by integrating polarization and spatial features. Fu et al. [23] utilized an optimized RF algorithm combined with multi-temporal SAR and optical imagery data to successfully improve the classification accuracy of wetland vegetation. Shao et al. [24] used RF and extreme gradient lift classifiers, combined with the integration of optical and SAR features and simple layering technology to classify impervious urban water surfaces. Van et al. [25] utilized RF classification model based on multi-source remote sensing data to generate an overall map of salt marsh vegetation extent and further produced detailed maps of specific salt marsh vegetation habitats.

The water index method constructs specific mathematical expressions to highlight the differences between water and non-water areas in SAR images, thereby enabling water body extraction. Du et al. [26] effectively extracted water body information around salt lakes using Sentinel-1 SAR data and a threshold segmentation method and found that the results of water extracted by using Sentinel-1 Dual-Polarized Water Index (SDWI) were superior to those using single-polarization images. Guo et al. [27] constructed SDWI based on Sentinel-1 data and combined multi-scale segmentation and object-oriented classification methods to successfully extract water from complex terrain, while eliminating the influence of buildings and mountains. Jia et al. [28] proposed a water extraction method based on the SDWI for large-scale water body identification using Sentinel-1 data equipped with C-band SAR.

It has been found in practical applications that there are limitations in extracting water bodies using a single method. The SDWI method performs well in water body extraction in small areas, but it is prone to interference from noise and complex terrain, resulting in misidentification. The RF method has strong adaptability to complex environments, but it easily produces misidentifications, leading to the extraction of small areas that are not water bodies. Aiming to resolve the problems of low accuracy and unstable results that arise in water body extraction from Sentinel-1 SAR images through a single method, this paper proposes a fusion method for deriving water body extraction results, combining the SDWI and RF algorithms. The structure of this paper is organized as follows: Section 2 introduces the water body extraction methods for SAR images, fusing SDWI and RF; Section 3 presents the study areas (Dalu Lake, Yinfu Reservoir, and Huashan Reservoir) and the remote sensing data used, along with their preprocessing; Section 4 evaluates the proposed method, conducting experiments on high-resolution images and analyzing the results; Section 5 provides a summary of the methods used in this paper and offers prospects for future work.

## 2. Water Body Extraction Method

### 2.1. Sentinel-1 Dual-Polarized Water Index

**Figure 1:**
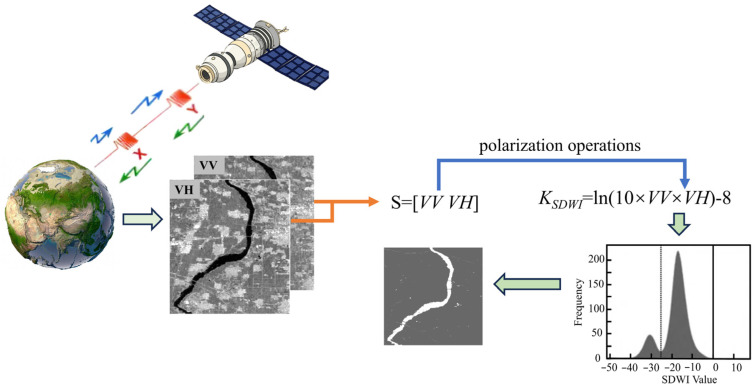
Schematic diagram of the polarization principle for the water index.

The SDWI is an advanced method specifically used for water body extraction in remote sensing imagery. This method is inspired by the Normalized Difference Vegetation Index (NDVI) and NDWI, and it incorporates the unique manifestation of water body information in imagery from microwave remote sensing technology. The core of this method lies in comprehensively utilizing the scattering characteristics of dual-polarization radar data—specifically the vertical transmit–vertical receive (VV) and vertical transmit–horizontal receive (VH) polarized backscattering coefficients—to enhance the separability between a water body and other land cover types; thereby, this approach enables effective water body extraction, as shown in Figure 1. The model of the SDWI is as follows:

(1) $$K_{\mathrm{SDWI}}=\ln(10\times VV\times VH)-8$$

where $K_{\mathrm{SDWI}}$ is a water body extraction index. When its value is greater than 0, it is a water body; when its value is less than 0, it is a non-water body. VV is the pixel value in the VV polarization channel of the SAR image, representing the backscatter intensity under the vertical transmit–vertical receive condition. VH is the pixel value in the VH polarization channel, representing the backscatter intensity under the vertical transmit–horizontal receive condition. This water index integrates dual-polarization information from VV and VH, offering higher accuracy compared to single-polarization threshold methods, and has been widely adopted in domestic applications. However, the empirical constant in the formula has strong subjectivity; the number of pixels corresponding to the bottom position in the histogram is large; the weak change in the threshold value will lead to a large change in the classification accuracy.

### 2.2. Random Forest Algorithm

The RF algorithm is a machine learning algorithm that integrates the predictions of individual decision trees through a voting mechanism (classification problem) or an averaging mechanism (regression problem). The basic principles of the RF algorithm are described here: ① Bagging is utilized to generate multiple training sample sets. Each training sample set randomly selects N samples from the original dataset (with retracted sampling) to form a new training sample set. Repeating the above steps T times, one can generate T different training sample sets. When the sample size is large, approximately one-third of the samples are not in the training dataset of any tree. Samples like this, that have not been extracted, are called Out of Bag (OOB) data. ② Each training subset is used to construct a decision tree. During the partitioning process of each node of the decision tree, not all features are used for partitioning. Instead, a feature subset of size M is randomly selected from the original feature set, and the optimal partitioning features and thresholds are sought on this subset. For classification problems, the principle of the minimum Gini index is used to select the optimal division. For regression problems, the principle of minimum variance is adopted for division. When a decision tree node splits, the definition formula of its Gini coefficient is as follows:

(2) $$Gini(T)=1-\sum_{i=1}^{K}pi^{2}$$

**Figure 2:**
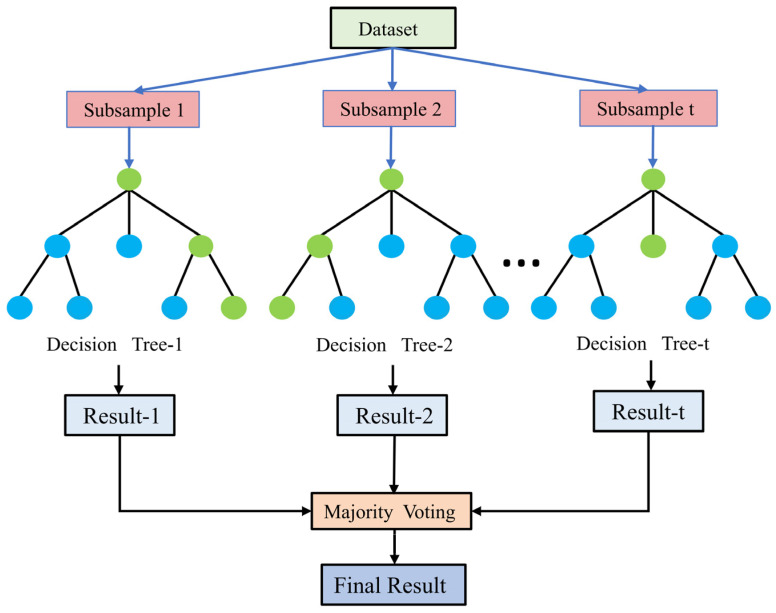
Random forest algorithm.

In the formula, T represents the sample set corresponding to the current node and $p_{i}$ represents the frequency of occurrence of class i in the sample set T. ③ The final prediction result is obtained by aggregating the predictions of all decision trees. The process is shown in Figure 2. The algorithm integrates multiple decision trees, and reduces the error of a single model, but due to its randomness, the results will fluctuate to a certain extent.

### 2.3. SDWI and RF Fusion Method

The SDWI and RF methods were used to extract water bodies from Sentinel-1 imagery, and morphological processing was carried out on the binary images obtained using this process, including corrosion, expansion, and filling operations. The principles of this process are described here.

(1) Corrosion is the minimum value of the difference between f and b in the area covered by structural element, b, for each pixel position, (x,y). The etching operation reduces the light areas in the image and enlarges the dark areas. Its expression is

(3) $$\left(f⊝b)(x,y)=\underset{(s,t)\in D_{b}}{\min}\{f(x+s,y+t)-b(s,t)\right\}$$

where f represents the function of the input image (grayscale image or binary image); b is the structural element, that is, the kernel in morphological operations (such as circle, cross, etc.), which is used to define the neighborhood; $D_{f}$ and $D_{b}$ are the domains of f and b, respectively. (x,y) is the pixel position in the image; (s,t) is the offset of structural element, b; (f⊝b) is the grayscale image after corrosion.

(2) Dilation is defined as calculating the maximum value of the difference between f and b within the region covered by the structuring element b for each pixel position, (x,y). The dilation operation expands the bright regions in the image and reduces the dark regions. Its mathematical expression is

(4) $$\left(f⊕b)(x,y)=\underset{(s,t)\in D_{b}}{\min}\{f(x-s,y-t)+b(s,t)\right\}$$

where (f⊕b) is the grayscale image after corrosion.

(3) The filling operation is used to fill holes within the foreground objects, and its mathematical expression is

(5) $$Fill(I,B)=\underset{n\to \infty }{\lim}{\left(I⊕B\right)}^{n}$$

where I is the input binary image, B is the structural element, ⊕ is the expansion operation, and n is the number of iterations until the filling operation converges (no longer changes); Fill(I,B) is the result image after filling.

(4) The results after morphological processing are, respectively, recorded as follows: $M_{\mathrm{NDWI}}$, $M_{\mathrm{SDWI}}$, and $M_{\mathrm{RF}}$. The intersection inversion operation with the reference mask $M_{\mathrm{NDWI}}$ is achieved through set operation, and the consistency constraint of the SDWI result mask is imposed. The expression is

(6) $$M_{\mathrm{SDWI}-\mathrm{Opt}}=M_{\mathrm{SDWI}}\backslash (M_{\mathrm{SDWI}}\cap M_{\mathrm{NDWI}})$$

In the formula, $M_{\mathrm{NDWI}}$ represents a high-confidence water mask and serves as a reliable reference standard for water body. $M_{\mathrm{SDWI}}$ represents the water mask after morphological processing with the SDWI method. $M_{\mathrm{SDWI}-\mathrm{Opt}}$ represents the optimized water mask. This step aims to eliminate the suspected misjudged regions that are inconsistent with the results of high-confidence NDWI and effectively suppress the possible false positives in the SDWI.

(5) The consistency constraints are imposed on the masks $M_{\mathrm{RF}}$ and $M_{\mathrm{NDWI}}$ of the RF classification results. The regions that are inconsistent with the high-confidence mask, $M_{\mathrm{NDWI}}$, are eliminated through the set difference operation. The expression is

(7) $$M_{\mathrm{RF}-\mathrm{Opt}}=M_{\mathrm{RF}}\backslash (M_{\mathrm{RF}}\cap M_{\mathrm{NDWI}})$$

In the formula, $M_{\mathrm{RF}}$ represents the water mask after morphological processing using the RF method. This step eliminates the regions in RF classification that have significant conflict with $M_{\mathrm{NDWI}}$, in order to reduce the impact of misclassification (especially false positives). The optimized mask $M_{\mathrm{RF}-\mathrm{Opt}}$ retains the ability of the RF to identify potential water body (such as shadow interference and blurred areas of the water—land boundary) in complex backgrounds. Meanwhile, it achieves result constraints with the help of NDWI information at the spatial consistency level, thereby improving the accuracy and reliability of the overall water body identification.

(6) The above-mentioned optimized masks are fused through logical operations to obtain the final water mask, and the expression is

(8) $$M_{\mathrm{Final}}=M_{\mathrm{SDWI}-\mathrm{Opt}}\cup M_{\mathrm{RF}-\mathrm{Opt}}$$

**Figure 3:**
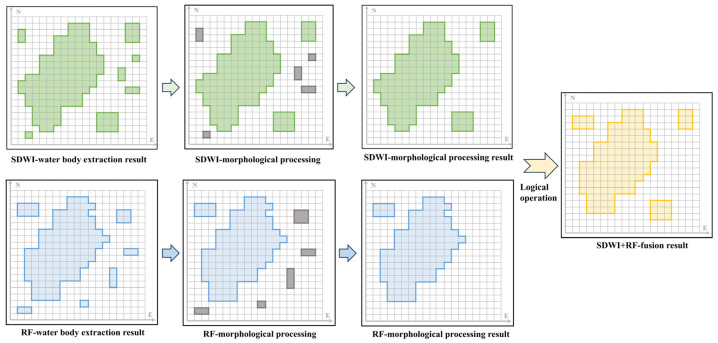
Fusion process diagram.

This strategy fully combines the sensitivity of SDWI to tiny water body and shadow areas with the robustness of the RF model in the discrimination of complex ground features. This fusion operation effectively controls the risk of false positives caused by union while ensuring the integrity of water body detection, thereby achieving a more accurate characterization of the spatial distribution of water body. The fusion process is shown in Figure 3.

## 3. Research Area and Data Sources

### 3.1. Research Area

**Figure 4:**
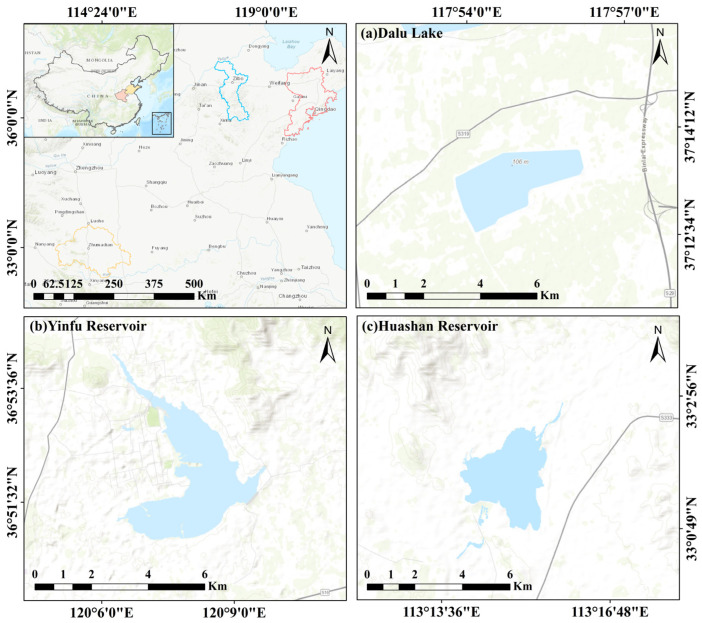
Study area diagram: (**a**) Dalu Lake research area; (**b**) Yinfu Reservoir research area; (**c**) Huashan Reservoir research area.

Dalu Lake is located in the east of Boshan District, Zibo City, Shandong Province, as shown in Figure 4a. Its geographical coordinates range from 117.9° to 118.1° east longitude and 36.5° to 36.7° north latitude. The lake belongs to the important water resources and ecological system in Shandong Province, and its geographical position is superior. The surrounding area is hilly, the vegetation around the lake is relatively rich, and the water system is relatively developed. The lake area varies with the seasons, with the water level usually higher in summer and lower in winter. There are many kinds of organisms in the lake area, which have certain ecological protection significance.

Yinfu Reservoir is located in the northeast of Pingdu City, Qingdao City, Shandong Province, as shown in Figure 4b. Its geographical coordinates are about 119°52′ east longitude and 36°46′ north latitude. The upper reaches of the reservoir cover several drainage basins in Pingdu City and its surrounding areas. The natural landscape around the reservoir is mainly dominated by hills and low mountains, and the terrain is undulating. The soil in the area where the reservoir is located is mainly sandy loam, and the reservoir has a strong water storage capacity, but it is also affected by seasonal precipitation changes. As a typical water resources area, it is of great significance to the study of water resources management, ecological environment protection, and flood regulation.

Huashan Reservoir is located in the southeast of Zhumadian City, Henan Province, as shown in Figure 4c. Its geographical coordinates range from approximately 114.0° to 114.2° east longitude and from 32.8° to 33.0° north latitude. As an important water source project in southern Henan, the reservoir plays a critical role in regional agricultural irrigation, urban water supply, and flood control. The surrounding terrain is mainly composed of low hills and gentle slopes, with relatively abundant vegetation coverage. The water body area of Huashan Reservoir shows clear seasonal variability, typically expanding during the rainy season and shrinking in dry periods. The aquatic and terrestrial ecosystems in the region are relatively diverse, and the reservoir serves important ecological and environmental protection functions in the local watershed system.

### 3.2. Data Sources

In order to extract the water bodies in the study areas, the experiment adopted the Sentinel-1 and Sentinel-2 surface reflectance products. The specific parameters of the data source are shown in Table 1.

The SAR image data are sourced from the Sentinel-1A satellite, which was launched by the European Space Agency (ESA) in 2014. It primarily operates in the C-band for Earth observation, with an orbital altitude of approximately 693 km and a revisit period of 12 days. The Sentinel-1 data provided by the Google Earth Engine (GEE, https://earthengine.google.com) platform have completed part of the preprocessing operations, with a resolution of 10 m, and were obtained using the VV and VH dual-polarization interference wide width (IW) mode. The images are cropped based on the study area, and filtering methods are applied to reduce speckle noise in the SAR images.

Sentinel-2 is an earth observation satellite system jointly developed by the European Space Agency (ESA) and the European Commission, consisting of two satellites, Sentinel-2A and Sentinel-2B. It provides high-resolution multispectral remote sensing data with global coverage and a revisit period of 5 days. The Sentinel-2 satellites are equipped with a Multispectral Instrument (MSI) featuring 13 spectral bands, including the visible and near-infrared bands. In this study, the remote sensing data used are those from the Sentinel-2 L2A product, obtained from the COPERNICUS/S2_SR dataset provided by GEE. This dataset has undergone Bottom-Of-Atmosphere (BOA) atmospheric correction, providing standardized surface reflectance products.

### 3.3. Experimental Process

**Figure 5:**
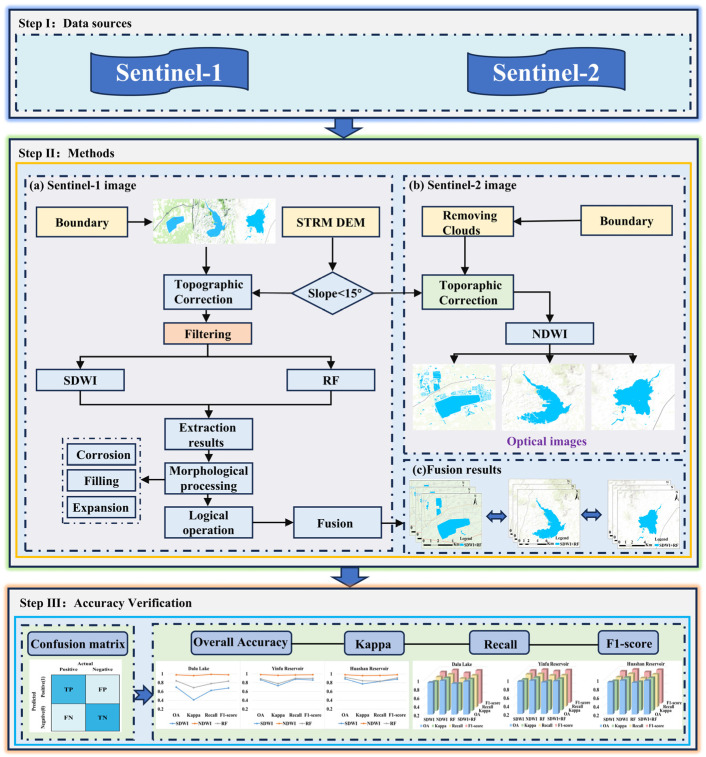
Water body extraction experiment flowchart.

Based on Sentinel-1 SAR images and Sentinel-2 optical images, the water bodies in the target area were extracted, compared, and analyzed using SDWI, RF, and NDWI, according to the following steps: (1) determine the study area; (2) select segmentation parameters; (3) preprocess images; (4) extract the water bodies using the SDWI, RF, and NDWI methods, respectively; (5) apply the morphological algorithms to process the extracted water bodies; (6) conduct the fusion and comparative analysis of results. The technical process is shown in Figure 5.

### 3.4. Precision Evaluation Index

In order to evaluate the accuracy of water body extraction in remote sensing images, a confusion matrix was first constructed. The confusion matrix shows the corresponding relationship between the classification result and the actual observation value, including the pixel number of correct classification and misclassification, which provides data support for the subsequent accuracy evaluation.

Based on the confusion matrix, four commonly used accuracy assessment metrics were used to comprehensively evaluate the water extraction results: overall accuracy (OA), recall, F1-score, and Kappa coefficient. The formulas are shown in Equations (9)–(13).

(1) Overall accuracy reflects the proportion of correctly classified pixels among all pixels, and its calculation formula is as follows:

(9) $$OA=\frac{TP+TN}{TP+TN+FP+FN}$$

In the formula, TP represents the number of pixels correctly identified as water body (actually water body, predicted as water body); TN represents the number of pixels correctly identified as non-water body (actual non-water body that has been predicted to be a non-water body); FP represents the number of non-water body pixels wrongly identified as water body pixels (actual non-water body that has been predicted to be a water body); FN represents the number of water body pixels wrongly identified as non-water body pixels (actual water body that has been predicted to be a non-water body).

(2) The producer’s accuracy represents the proportion of correctly extracted pixels in the actual category, and its calculation formula is as follows:

(10) $$Recall=\frac{TP}{TP+FN}$$

(3) The F1-score is the harmonic mean of the precision and the recall, combining the performance of the classifier in both positive and negative classes. Its calculation formula is as follows:

(11) $$F1\text{-}score=\frac{2\times Precision\times Recall}{Precision+Recall}$$

(4) The Kappa coefficient is used to evaluate the agreement between the classification results and the random classification results. Its calculation formula is as follows:

(12) $$Kappa=\frac{OA-p_{e}}{1-p_{e}}$$

where the expression of $p_{e}$ is

(13) $$p_{e}=\frac{N_{1}R_{1}+N_{2}R_{2}}{C}$$

where $N_{1}$ indicates that the extraction result is the water region; $N_{2}$ indicates that the extraction result is the non-water area; $R_{1}$ stands for the water area; $R_{2}$ represents the non-water area; C indicates all areas.

## 4. Result Analysis and Verification

### 4.1. Water Body Extraction Results and Analysis

**Figure 6:**
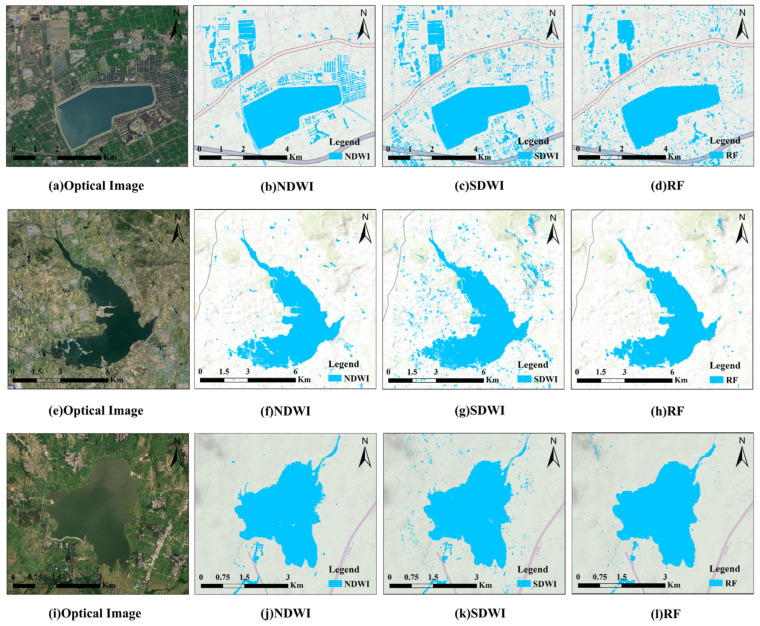
(**a**,**e**,**i**) Optical image diagrams; (**b**,**f**,**j**) extraction results using the NDWI method; (**c**,**g**,**k**) extraction results using the SDWI method; (**d**,**h**,**l**) extraction results using the RF method.

On the Sentinel-2 optical images, NDWI was applied to extract the water body. On Sentinel-1 SAR images, the SDWI and RF classification methods were adopted to extract water body information. By comparing the matching degree of water body results extracted by different methods with those extracted by NDWI method and optical images, the effectiveness and reliability of each method in the water body extraction process were verified. The water body extraction results are shown in Figure 6. In the study areas of Dalu Lake, Yinfu Reservoir, and Huashan Reservoir, 600 sample points were selected for each area, with 70% used for training and approximately 30% for testing. By calculating the confusion matrix, commonly used accuracy evaluation indexes were obtained to comprehensively evaluate the results of water body extraction.

(1) NDWI was used to extract water body from Sentinel-2 images and the results are shown in Figure 6b,f,j. Compared with the optical images, it can be clearly seen that the water body regions are accurately and continuously identified, showing high consistency with the water body distribution in the original images. Moreover, NDWI can accurately identify small water bodies, indicating that this method has high sensitivity in water body extraction. According to the statistical results in Table 2, the overall accuracy of the three study areas reached 97.9%, 98.4%, and 98.3%, respectively, showing the high precision and reliability of the method in water extraction.
(2) Figure 6c,g,k show the results of the water body extraction process from the SAR images using the SDWI. Compared with Figure 6a,e,i, the water bodies extracted by the SDWI method have a large error. This is mainly due to the fact that SAR images are susceptible to noise interference, resulting in some non-water-body areas being misjudged as water body areas. According to the statistical data in Table 2, the overall accuracies of the SDWI method in the three study areas are 70%, 86.6%, and 86.8%, respectively, which are significantly lower than those derived using the NDWI method. However, the SDWI demonstrates higher precision in identifying small water bodies, effectively distinguishing smaller water areas.
(3) Figure 6d,h,l show the water body results extracted using the RF algorithm. Compared with optical images, the RF algorithm has less error in the recognition of large-area water bodies. However, there are obvious errors in the extraction of small water bodies, especially in the extraction of small water bodies such as ponds and irrigation canals, and the classification results are not ideal.

**Table 2:** Water body extraction accuracy.

Areas	Methods	OA (%)	Kappa (%)	Recall (%)	F1-Score (%)
Dalu Lake	NDWI	0.979	0.961	0.989	0.980
	SDWI	0.700	0.410	0.625	0.679
	RF	0.843	0.688	0.779	0.835
Yinfu Reservoir	NDWI	0.984	0.968	0.976	0.981
	SDWI	0.866	0.732	0.881	0.860
	RF	0.890	0.779	0.898	0.898
Huashan Reservoir	NDWI	0.983	0.962	0.965	0.982
	SDWI	0.886	0.773	0.844	0.880
	RF	0.924	0.846	0.835	0.910

### 4.2. Morphological Processing and Fusion Results

**Figure 7:**
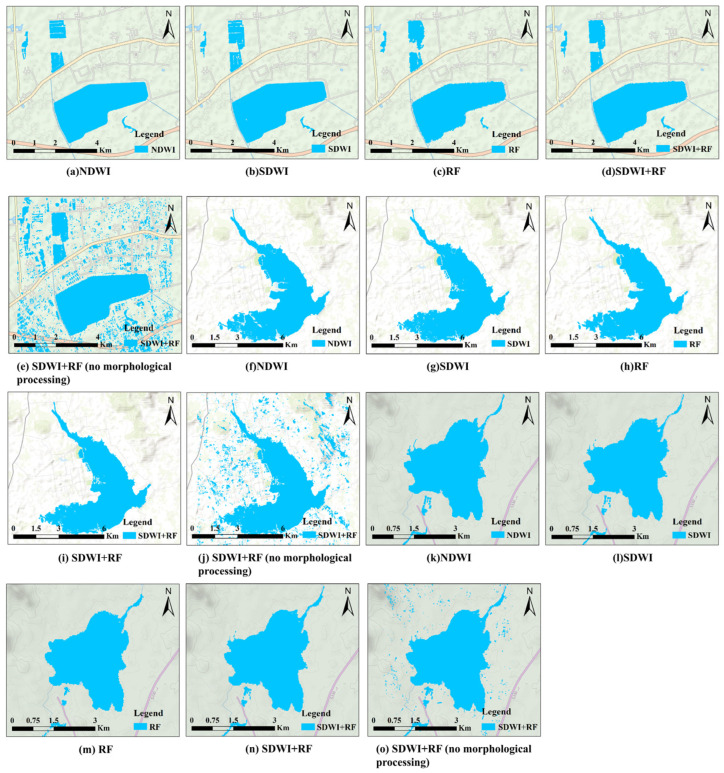
Comparison of morphological processing and fusion.

In the results extracted using the SDWI and RF methods, there is a significant occurrence of non-water areas being misclassified as water bodies, which interferes with the water body extraction results. Therefore, morphological processing was applied to the water bodies extracted using the above methods, and the processed results are shown in Figure 7. The water body extraction results were further refined, ultimately obtaining the fused water body results from SDWI and RF, as shown in Figure 7d,e,i,j,n,o. These results were then compared with the water body extraction results from other methods. The accuracy comparison is shown in Table 3; the analysis of the results is presented here.

(1) Figure 7a,f,k illustrate the water body extraction results derived from the NDWI applied to Sentinel-2 optical images. The NDWI leverages the spectral characteristic differences between the green and near-infrared bands to effectively suppress the interference from non-water-body features (such as vegetation and soil), while enhancing water body characteristics. When comparing the water body extracted using this method with the original optical imagery, the overall accuracies in the three study areas are 98.4%, 98.5%, and 98.4%, respectively, demonstrating high precision and stability.
(2) The SDWI-based water body extraction results from SAR images exhibit noticeable misclassifications; as shown in Figure 7b,g,l, there are numerous blue spots in non-water areas (misidentified as water bodies) and white spots in water body areas (misidentified as non-water bodies). At the same time, there are obvious misidentification phenomena. Although the SDWI method can clearly identify small water bodies, and the overall accuracy is more than 94%, as shown in Table 3, its sensitivity to noise and limitations in complex environments significantly affect the accuracy of water body extraction.
(3) The water extraction results obtained using RF with morphological post-processing are illustrated in Figure 7c,h,m. The RF method exhibits strong adaptability to complex environments and achieves high accuracy in identifying extensive water bodies. However, the algorithm shows limitations when applied to small or fragmented water bodies, which are often misclassified as non-water bodies due to their limited spatial continuity and weak scattering signatures. In the interleaving areas of water bodies and non-water bodies, the backscattering characteristics of small non-water areas (such as shallow banks, bare soil, or areas with low vegetation cover) are similar to those of neighboring water bodies, and these are easily misidentified as water bodies. There are some local errors in the extraction results.
(4) Figure 7d,i,n show the water results extracted by fusing the SDWI and RF methods. Compared with the SDWI alone, the fusion derives results that effectively reduce the large area of misidentification, especially in the relatively simple water body area. The fusion method makes full use of the advantages of the RF algorithm in large-scale water body identification and avoids the sensitivity of the SDWI method to noise and complex environments. In addition, compared with the RF method alone, the fusion results were effectively supplemented in the area of unidentified small water bodies, effectively making up for the shortcomings of the single method and improving the accuracy of the overall extraction.
(5) Figure 7e,j,o present the results of the direct fusion of the SDWI and RF extraction results without morphological processing. This direct fusion can, to some extent, supplement the recognition differences of a single algorithm, such as filling in the tiny water body missed by RF; however, due to the fact that the original results of the SDWI are easily interfered with by SAR speckle noise, “false water patches” are generated in areas such as building shadows and bare soil, resulting in superposition, retention of noise, and misjudgment. This leads to a dense concentration of false water body patches in the fusion results, seriously interfering with the boundaries and scope of the real water bodies and resulting in a significant deviation of the water body results from reality.

**Table 3:** Precision comparison results.

Areas	Methods	OA (%)	Kappa (%)	Recall (%)	F1-Score (%)
Dalu Lake	NDWI	0.984	0.968	0.965	0.981
	SDWI	0.942	0.882	0.903	0.933
	RF	0.917	0.833	0.868	0.915
	SDWI + RF	0.969	0.939	0.941	0.970
	SDWI + RF (no morphological processing)	0.685	0.371	0.593	0.654
Yinfu Reservoir	NDWI	0.985	0.969	0.968	0.982
	SDWI	0.963	0.925	0.961	0.958
	RF	0.945	0.890	0.933	0.949
	SDWI + RF	0.972	0.943	0.938	0.971
	SDWI + RF (no morphological processing)	0.853	0.706	0.893	0.856
Huashan Reservoir	NDWI	0.984	0.969	0.971	0.985
	SDWI	0.945	0.891	0.893	0.943
	RF	0.934	0.869	0.871	0.927
	SDWI + RF	0.981	0.961	0.958	0.978
	SDWI + RF (no morphological processing)	0.868	0.738	0.900	0.873

## 5. Conclusions

A fusion method for extracting water bodies from the Sentinel-1 dataset, using the SDWI and RF algorithms, is proposed; the aim of the fusion is to overcome the problems of low extraction accuracy and poor adaptability that arise in different environments when using a single method. By integrating the water body extraction results of two different algorithms, the possible deviation caused by a single method has been effectively reduced, the refinement degree of water body extraction has been significantly improved, and the accuracy of the extraction results has been enhanced. In addition, this study also explored the performance of SAR and optical images in water body extraction and compared the differences of different remote sensing data sources in practical applications. The results of Dalu Lake, Yinfu Reservoir, and Huashan Reservoir show that this fusion method is superior to the single method, demonstrating its accuracy and stability in different regions. Future work will further expand the adaptability of the algorithm, support the fusion of high-resolution and multi-temporal images, and is expected to further enhance the timeliness and accuracy of water body extraction, providing strong support for large-scale monitoring and dynamic change analysis.

## Figures and Tables

**Table 1:** Remote sensing satellite data parameters.

Data Sets	Date	Spatial Resolution	Polarization Mode/Band
Sentinel-1	1 October 2023–1 December 2023	10 m × 10 m	VV and VH
Sentinel-2	1 October 2023–1 December 2023	10 m × 10 m	B3 and B8

## Data Availability

Data are contained within the article.
